# Hemolytic Uremic Syndrome Incidence in New York**^1^**

**DOI:** 10.3201/eid1005.030456

**Published:** 2004-05

**Authors:** Hwa-Gan H. Chang, Boldtsetseg Tserenpuntsag, Marilyn Kacica, Perry F. Smith, Dale L. Morse

**Affiliations:** * New York State Department of Health, Albany, New York; † University at Albany, Albany, New York

**Keywords:** hemolytic uremic syndrome, Escherichia coli 0157:H7, surveillance, capture-recapture, sensitivity

## Abstract

A comparison of New York’s traditional communicable disease surveillance system for diarrhea-associated hemolytic uremic syndrome with hospital discharge data showed a sensitivity of 65%. Escherichia coli O157:H7 was found in 63% of samples cultured from hemolytic uremic syndrome patients, and samples were more likely to be positive when collected early in illness.

Diarrhea-associated hemolytic uremic syndrome (HUS) is a major cause of acute renal failure in children (*1*). Various studies have demonstrated that Shiga toxin–producing *Escherichia coli* are the etiologic agents in most cases of diarrhea-associated HUS (*2*,*3*). In the United States, *E. coli* O157:H7 is the most common Shiga toxin–producing *E. coli*, causing an estimated 73,480 infections (*4*); HUS develops in 3%–15% of patients soon after the onset of diarrhea (*5*,*6*).

The risk factors associated with the progression of *E. coli* O157 to HUS include very young or old age, elevated leukocyte count, and use of antimicrobial treatment or antimotility agents (*7*–*11*). Little is known about the clinical features that could identify patients at high risk for HUS. In New York, both *E. coli* O157 infection and diarrhea-associated HUS became reportable in July 1994. Since then, the annual incidence rate for *E. coli* O157 per 100,000 population has ranged from 1.6 in 1995 to 8.5 in 1999; the annual incidence rate for diarrhea-associated HUS per 100,000 population has ranged from 0.1 in 1995 to 0.2 in 1999.

HUS is a severe disease that results in hospitalization. Our surveillance and hospital discharge data were used to evaluate the HUS surveillance system, estimate the number of diarrhea-associated HUS cases, and study the epidemiologic and clinical features of HUS in New York.

## The Study

A confirmed case of HUS was defined as the acute onset of anemia with microangiopathic changes (i.e., schistocytes, burr cells, or helmet cells) on peripheral blood smear, acute renal injury (i.e., hematuria, proteinuria, or elevated creatinine level), and a low platelet count (platelets <150,000/mL) within 3 weeks of an acute diarrheal illness. A probable case of HUS was defined as the acute onset of anemia with microangiopathic changes, acute renal injury, and a low platelet count without a history of diarrhea in the preceding 3 weeks; or hemolytic anemia without confirmed microangiopathic changes, acute renal injury, and a low platelet count within 3 weeks of an acute diarrheal illness (*12*).

Hospital admission notes, laboratory reports, and discharge summaries were requested for patients who had HUS (International Classification of Diseases, 9th Revision, Clinical Modification [ICD-9-CM] code 283.11) listed as their primary or any secondary diagnosis for 1998 and 1999. All medical charts were reviewed, and patients were classified as having a confirmed, probable, or undetermined HUS case. Persons with multiple hospitalizations were counted only once. The records from case reports and medical charts were matched by last name, first name, and date of birth. The capture-recapture method was used to evaluate the completeness of HUS reporting and to estimate the “true” number of HUS cases and the 95% confidence interval (*13*).

Data from medical charts regarding demographic characteristics (age, sex, race, month of admission, hospital length of stay), clinical features at admission (vomiting, fever, bloody stool), and antimicrobial therapy were extracted for all confirmed or probable case-patients. Laboratory variables were obtained for patients within 7 days before and 3 days after the HUS diagnosis and included the lowest hematocrit, lowest platelet count, highest blood urea nitrogen (BUN), and highest creatinine concentrations. The Fisher exact test was used to determine the proportion of demographic and clinical characteristics among HUS patients with or without stool isolates for *E. coli* O157:H7, and the two-tailed Student *t* test was used to determine the significance of differences between their mean laboratory variables.

Forty-five HUS case-patients reported to the New York communicable disease surveillance system for 1998 through 1999 were listed as being hospitalized; the medical charts of 44 of these patients were available for review. We requested 542 medical records that had a primary or secondary discharge diagnosis listed as HUS during the same period; 421 (78%) charts were received. After chart review, 234 records were from New York State residents, excluding New York City, and 201 patients remained for analysis after excluding duplicate records. Forty-nine patients had confirmed HUS, 10 patients had probable HUS, and 142 patients either had incomplete information to determine HUS status or did not have HUS. Among these 142 patients, 9 patients had no laboratory reports, 23 patients had renal failure and anemia with microangiopathic changes but no diarrhea; and 110 patients had no renal failure or no microangiopathic changes. Stool samples from 12 of these 142 patients were cultured for *E. coli*; none was positive.

HUS surveillance data were matched against hospital discharge data by last name, first name, and date of birth. Thirty-three confirmed and 5 probable cases were in both systems, 4 confirmed and 3 probable cases were only in the surveillance system, and 16 confirmed and 5 probable cases were only in the hospital system. The “true” total number of confirmed or probable HUS cases in New York was estimated to be 70 (95% confidence interval [CI] = 63 to 75), and the sensitivity of our surveillance system for reporting HUS was 65%.

The demographic, clinical features, and laboratory results of 53 confirmed and 12 probable diarrhea-associated HUS cases from either data system were studied. The average annual incidence was significantly greater among children <5 years of age (relative risk [RR] 17, 95% confidence interval [CI] 8.9 to 32) than among patients 15–64 years of age and was greater among female than male patients (RR 2.1, 95% CI 1.2 to 3.8) (Table 1). The median length of hospital stay was 11 days. Review of clinical features indicated that 62 (95%) had diarrhea; 46 (71%) had bloody stools. While hospitalized, 20 (31%) patients received antimicrobial agents and 19 were treated before HUS developed. Thrombocytopenia (platelets <150,000/mL) was present in 90% of the patients, and 85% had hemolytic anemia with microangiopathic changes on peripheral blood smear. HUS patients <15 years of age were more likely to have fever (64%) and bloody stools (82%) than those >15 years of age (19% and 54%, respectively).

**Table 1:** Demographic and clinical characteristics of 65 patients with confirmed or probable hemolytic uremic syndrome (HUS), New York, 1998–1999

Characteristic	No. of patients (N = 65)
Demographic features	No. (incidence/100,000)
Age (in years)	
<5	28 (2.0)
5–14	11 (0.4)
15–64	17 (0.1)
>65	9 (0.3)
Sex	
Male	20 (0.2)
Female	45 (0.4)
Race	
White	57 (0.3)
Black	3 (0.2)
Asian	1 (0.2)
Outcome	No. (%)
Alive	59 (91)
Dead	6 (9)
Outbreak-associated	No. (%)
Yes	15 (23)
No	50 (77)

Six patients (9%), ages 3 to 89 years, died while hospitalized; two of these deaths were outbreak related. All six patients had bloody diarrhea and blood transfusions, leukocyte counts >18,000, BUN levels >63, and serum creatinine levels >2.3. Samples from five of six were culture positive for *E. coli* O157:H7.

Of the 65 confirmed or probable HUS patients, 54 (83%) had their stool or urine tested for *E. coli* O157:H7; 34 (63%) of these patients had positive results (33 stools and 1 urine). Of the 34 culture-confirmed patients, 30 were reported to the surveillance system, and 4 patients received a diagnosis only in the hospital system. A significantly higher proportion of patients with samples culture-positive for *E. coli* O157 patients had shorter mean durations from diarrhea onset to specimen collection, and lower BUN values within 7 days of admission than did culture-negative *E. coli* O157 patients (Table 2). A higher proportion of patients with outbreak-related HUS cases had positive *E. coli* O157 cultures (13/15, 87%) than those with nonoutbreak-related cases (21/50, 42%).

**Table 2:** Characteristics of HUS cases by *E. coli* O157:H7 culture isolation status, New York, 1998–1999^a^

Characteristics	*Escherichia coli* O157:H7 culture		
	Positive (n = 34)	Negative (n = 20)	p value
Mean age at admission (y)	23.6	23.7	0.99
Mean length of hospital stay (days)	13.4	12.6	0.71
Mean duration from diarrhea onset to specimen collection date (days)	4.1	6.0	0.06
Median duration from admission date to specimen collection date (days)	0	1.0	0.02
Mean BUN (mg/dL)	62.1	82.6	0.05
Mean creatinine (mg/dL)	3.6	4.8	0.24
Mean platelet count (/10^3^)	50.9	53.8	0.81
Mean leukocyte count (/10^3^)	18.5	21.4	0.23
% outbreak related	38%	5%	0.01
% blood in stool	82%	65%	0.15
% reported to surveillance	88%	55%	0.01
% microangiopathic change	79%	100%	0.03
% urinary tract infection	3%	20%	0.04

^a^HUS, hemolytic uremic syndrome; *E. coli, Escherichia coli*; BUN, blood urea nitrogen.

## Conclusions

Most surveillance systems for communicable disease reporting are passive. The use of hospital discharge records is a conventional method to verify the completeness of reporting. A population-based study that used hospital data without reviewing medical charts estimated that 47% of hospital discharge data were reported to public health surveillance (*14*). Our disease surveillance system’s sensitivity of 65% in identifying diarrhea-associated HUS was higher than that figure but also reinforces the importance of medical chart audit. A possible contributing reason for the low sensitivity may be the difficulty in identifying confirmed HUS cases. To enhance surveillance, New York, as a FoodNet site, extended the chart review for hospitalized patients <18 years of age statewide in whom HUS was diagnosed.

Seven of the 12 probable HUS case-patients had *E. coli* O157:H7 isolated without evidence of erythrocyte fragmentation, suggesting that hospitals may not report probable cases. A study to match *E. coli* O157:H7 cases from our surveillance system against HUS diagnosed cases from a hospital discharge data system and to review all case-patients hospitalized with *E.* c*oli* O157:H7 is under way. Results should identify the possible diarrhea-associated HUS cases missed by our surveillance system.

This study showed that female patients had higher incidence rates for HUS; the incidence rates of *E. coli* O157:H7 showed no differences by gender. *E. coli* O157:H7 was isolated from stool or urine samples from 34 (63%) of 54 confirmed and probable HUS patients who were cultured, a finding that demonstrates that *E. coli* O157:H7 is the predominant organism related to diarrhea-associated HUS in New York. Other studies have had similar findings (*15*,*16*). Samples from HUS patients cultured on or before the hospital admission date had a higher recovery rate of *E. coli* O157 from stool cultures (78%) than those cultured after hospital admission (41%). Patients who did not have *E. coli* O157:H7 isolated were cultured later in illness (6 days) than those from whom this pathogen was recovered (4 days), a finding that reemphasizes the importance of obtaining early cultures for microbiologic diagnosis (*17*).

HUS associated with *E. coli* O157:H7 infection is a serious illness with a 5%–10% death rate (*5*). Prompt followup of cases using pulsed-field gel electrophoresis molecular methods has increasingly led to identifying food sources and removing contaminated products from the food distribution system. Complete and rapid reporting of cases is a crucial component of public health prevention activities.
